# Prediction of Individual User’s Dynamic Ranges of EEG Features from Resting-State EEG Data for Evaluating Their Suitability for Passive Brain–Computer Interface Applications

**DOI:** 10.3390/s20040988

**Published:** 2020-02-12

**Authors:** Ho-Seung Cha, Chang-Hee Han, Chang-Hwan Im

**Affiliations:** Department of Biomedical Engineering, Hanyang University, Seoul 04763, Korea; chayo89@hanyang.ac.kr (H.-S.C.); zeros8706@naver.com (C.-H.H.)

**Keywords:** electroencephalography (EEG), interindividual variability, resting state EEG, passive brain–computer interfaces, machine learning

## Abstract

With the recent development of low-cost wearable electroencephalogram (EEG) recording systems, passive brain–computer interface (pBCI) applications are being actively studied for a variety of application areas, such as education, entertainment, and healthcare. Various EEG features have been employed for the implementation of pBCI applications; however, it is frequently reported that some individuals have difficulty fully enjoying the pBCI applications because the dynamic ranges of their EEG features (i.e., its amplitude variability over time) were too small to be used in the practical applications. Conducting preliminary experiments to search for the individualized EEG features associated with different mental states can partly circumvent this issue; however, these time-consuming experiments were not necessary for the majority of users whose dynamic ranges of EEG features are large enough to be used for pBCI applications. In this study, we tried to predict an individual user’s dynamic ranges of the EEG features that are most widely employed for pBCI applications from resting-state EEG (RS-EEG), with the ultimate goal of identifying individuals who might need additional calibration to become suitable for the pBCI applications. We employed a machine learning-based regression model to predict the dynamic ranges of three widely used EEG features known to be associated with the brain states of valence, relaxation, and concentration. Our results showed that the dynamic ranges of EEG features could be predicted with normalized root mean squared errors of 0.2323, 0.1820, and 0.1562, respectively, demonstrating the possibility of predicting the dynamic ranges of the EEG features for pBCI applications using short resting EEG data.

## 1. Introduction

Brain–computer interface (BCI) is an emerging technology that helps decode neural signals for controlling external devices or communicating with others [1]. The technology has been implemented using various brain imaging methods, including electroencephalogram (EEG), magnetoencephalography (MEG), functional magnetic resonance imaging (fMRI), and functional near-infrared spectroscopy (fNIRS). Among these, EEG has been the one most widely used because of its noninvasiveness, high temporal resolution, portability, and reasonable cost [2].

BCI can be roughly categorized into two large areas: active BCI (aBCI) and passive BCI (pBCI). aBCI interprets the user’s intentions from self-modulated neural signals, which can be used to control external devices, such as wheelchairs and computers. On the other hand, pBCI monitors the user’s spontaneous neural signals. The neural signals can be correlated with cognitive or affective states of the user, and an appropriate feedback can sometimes be provided depending on the applications [3]. Over the past decades, many aBCI studies have been conducted for exploring novel methods of communication (e.g., mental speller, brain switch system, and robotic arm controller) in patients with locked-in syndrome [2]. Meanwhile, with the recent development of wearable EEG recording devices [4,5], increasing interest has been drawn toward pBCI applications in practical and daily-life scenarios [6,7,8].

When users conduct a specific task in the pBCI application, specific task-related EEG patterns are generated; therefore, the selection of appropriate EEG features to extract the patterns is of importance. There have been well-established EEG features that are known to be closely associated with an individual’s mental states. Examples of these EEG features include frontal alpha asymmetry (FAA) that is modulated by emotional tasks [9]; frontal theta power (FTP) that is modulated by concentrative meditation [10]; frontal low beta power (FLBP) that is modulated by intensive tasks [11]; and prefrontal gamma power that reflects the stress level of a user [12]. However, it is frequently reported that some individuals have difficulty in fully utilizing the pBCI applications because the dynamic ranges of their EEG features (i.e., its amplitude variability over time) were too small to be used in the practical applications [13,14,15,16,17]. One potential method to tackle this issue involves finding personalized EEG features most appropriate for each user by conducting additional experiments before deploying the pBCI applications [18,19,20]. This type of calibration might improve the performance of the pBCI applications, especially for those who show smaller dynamic ranges of EEG features; however, it is inefficient to apply the calibration to all the users because those who exhibit EEG dynamic ranges large enough to be used for pBCI applications may not need the calibration. Indeed, in the previous studies [18,19,20], it took at least 15 min to acquire the EEG data for the calibration. If a user’s dynamic range of EEG features can be precisely predicted before calibration, with short resting EEG (e.g., 60 s in this study), these time-consuming calibration procedures can be skipped for the users who are expected to show sufficiently large dynamic ranges. 

In this study, we predict individual user’s dynamic ranges of the three EEG features that have been widely used to estimate valence, relaxation, and concentration in various pBCI applications from 60-s resting-state EEG (RS-EEG), with the ultimate goal being the identification of individuals who might need additional calibration. We employed a machine learning-based regression model to predict the dynamic ranges of the EEG features and compared their performances in terms of the predictive error.

## 2. Materials and Methods

### 2.1. Participants

Thirty-five participants (22 male and 13 female college students with ages 24.74 ± 1.59 years) volunteered to participate in our experiment. No participant reported any serious health problems, such as mental disorders or eye diseases, that might affect the experiments. Before the experiments, each participant was provided with a detailed explanation of the experimental protocols and signed a written consent. The participants received monetary compensation for their participation in the experiments. The study protocol was approved by the Institutional Review Board (IRB) of Hanyang University (IRB No. HYI-14-167-11).

### 2.2. Experimental Design

All EEG data were measured from two scalp electrodes placed at Fp1 and Fp2 sites with a commercial biosignal recording system (Active Two; Biosemi B.V., Amsterdam, the Netherlands). Reference and ground electrodes were attached to the left and right mastoids, respectively. The electrode locations of Fp1 and Fp2 were employed considering widely-used commercial wearable EEG devices with prefrontal channels [4], e.g., Mindset (NeuroSky, San Jose, CA, USA), Brainband (MyndPlay, London, UK), XWave (PLX devices, San Jose, CA, USA), and Neural Impulse Actuator (OCZ Technology, San Jose, CA, USA). The sampling rate was set at 2048 Hz.

**Figure 1:**
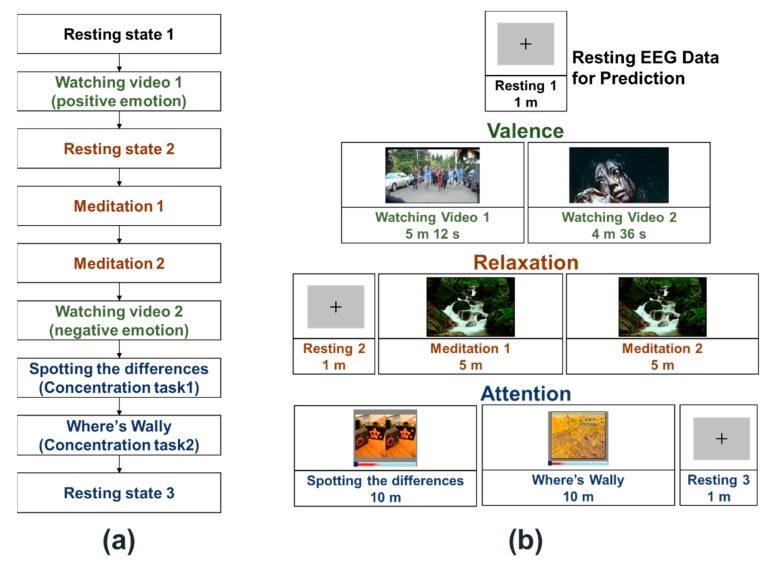
(**a**) Overall experiment procedures. (**b**) Overall structure of the three electroencephalogram (EEG) datasets recorded for each participant. Three experiments were designed to evoke different emotional states, make the participants relaxed, and elevate attention levels.

The overall experimental procedure is presented in Figure 1a. We designed the experimental protocol such that the three EEG features (valence, relaxation, and concentration) were investigated in a single experiment. Two different video clips were played to induce positive and negative emotions while recording the EEG signals, with the video clip that aroused positive emotion being played first. Experimental sessions investigating relaxation-related EEG features (denoted by meditation sessions) were added between the plays of positive and negative video clips as negative videos can potentially influence the following meditation sessions because of their contents being too intense for some participants. The sessions related to attention were allocated at the end of the experimental procedure because attention is relatively less dependent than meditation on the valence of the participants. 

Each participant sat in a comfortable chair in front of an LCD monitor and performed the designated experimental procedure according to the verbal instructions provided by the researcher. All the stimuli were presented using E-prime 2.0 software (Psychology Software Tools, Sharpsburg, PA, USA). 

Figure 1b shows the overall structure of the three datasets acquired from the experimental protocol presented in Figure 1b, where the text colors in Figure 1b match those in Figure 1a. The RS-EEG datasets (denoted by resting 1, resting 2, and resting 3 in Figure 1b) were recorded while the participants were staring at a fixed cross-mark displayed in the center of the monitor for a minute. Among the three RS-EEG datasets, the first one recorded during the resting 1 session was used to predict the EEG dynamic ranges.

Two video clips were played to induce positive and negative emotions. First, a 309-s-long clip titled “Isaac’s Live Lip-Dub Proposal [21]” was used to elicit a positive (happy) emotion, in which a man is seen proposing to his girlfriend. In the next session, a 276-s-long clip excerpted from a horror movie titled “JU-ON: THE GRUDGE 2 (2003) [22],” in which a woman is attacked by a ghost, was used to elicit a negative (scary) emotion. As already described earlier, the two sessions were allocated apart in the overall experiment procedure.

In the “meditation” sessions, the picture of a beautiful valley was presented on the monitor along with a seamless sound of flowing spring water. Simultaneously, a quiet periodic beep sound was provided to the participants at 3 s intervals. The participants were instructed to gently breathe in pace with the beep sound.

In the “attention” sessions, two different paradigms were adopted to elevate the attention level of the participants. In the first, the participants were instructed to visually search for differences between two similar images taken from the game “Spotting Differences”. The actual number of differences between the two images was five, but the participants were notified to look for six differences to make them continue searching for differences even after finding all five differences. Each participant was given 45 s for each picture, and this process was repeated for ten images. To make the participants maintain their attention during the session, the participants were notified that they should submit the answers after the end of the experimental session and would receive incentives if they found more than 45 different spots. In the second session, following an almost similar paradigm as the first, the participants were asked to visually search for the location of ‘Wally’ in the famous game “Where’s Wally?”. In this session, only 14 s were given to each participant, and the number of Wally’s locations was not notified to the participants. This trial was also repeated ten times. The participants were again notified that they should submit the answers after the end of the session and would receive incentives if they found more locations than the other participants. After the 1-min resting session that followed the two ”attention” sessions, the participants were asked to submit their answers to the experimenter to check whether they had accomplished the given task properly, and additional monetary reward was given to those participants who performed the task very well. We confirmed that all the participants paid good attention during the experiments. 

### 2.3. Overall Data Analysis Procedure

**Figure 2:**
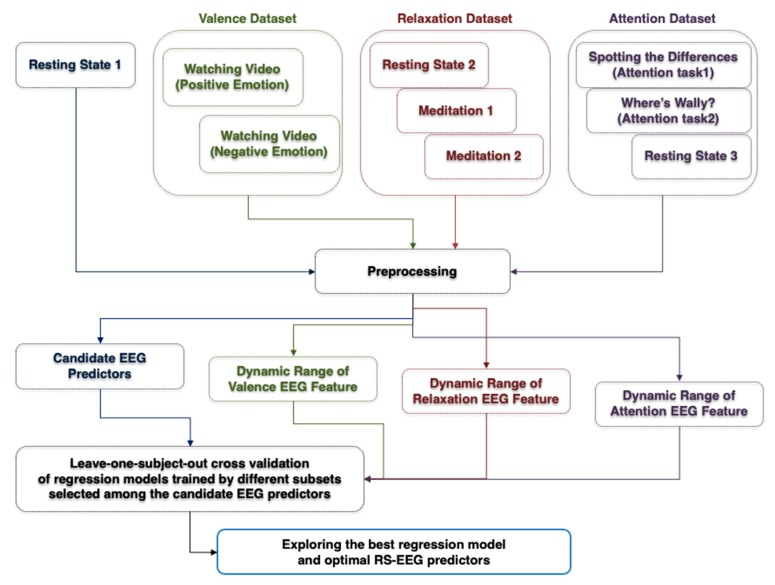
Flow chart of the overall data analysis process. Please note that text, text boxes, and arrows are displayed in different colors depending on the type of signal processing.

Figure 2 shows a flow chart showing the overall data analysis procedure. Four EEG datasets were used, including the first RS-EEG dataset (denoted by resting-state 1), the valence dataset, the relaxation dataset, and the attention dataset. The same preprocessing steps were applied to the four EEG datasets except for the attention dataset (the details are described in Section 2.4). After preprocessing, a number of candidate predictors of EEG dynamic ranges were evaluated from the preprocessed “resting state 1” dataset. The actual EEG dynamic ranges of each participant during different mental conditions were evaluated from his/her valence, relaxation, and attention datasets. Finally, a variety of regression models were constructed using different features selected among the candidate RS-EEG predictors and their performances were compared using the leave-one-participant-out cross-validation (LOOCV) scheme, resulting in the best regression model and the optimal RS-EEG predictors. The detailed signal processing procedures are explained in the following sections (Section 2.4, Section 2.5, Section 2.6 and Section 2.7).

### 2.4. Preprocessing

For the dataset including the first RS-EEG dataset, the valence dataset, and the relaxation dataset, raw EEG data were down-sampled to 256 Hz, and time periods with eye blink artifacts were detected using the multiwindow summation of derivatives within a window (MSDW) algorithm [23]. The down-sampled EEG data were bandpass-filtered at 0.5–30 Hz, and then segmented into a series of short segments using a sliding window of 1 s length with an overlap of 50%. Segments that were contaminated by eye blink artifacts were discarded.

For the attention dataset, ocular artifacts were removed using least-mean square (LMS)-based adaptive filtering [24] with two-channel EOG signals acquired around the right eyes (below and right of the right eye), instead of the MSDW algorithm. The LMS filtering was not applied to the valence and relaxation datasets because the EEG signals in these datasets were not severely contaminated by the eye saccadic artifacts. Therefore, the MSDW algorithm that does not require any additional EOG signal could sufficiently remove ocular artifacts.

### 2.5. Computing the Variability of the EEG Features 

In this study, frontal alpha asymmetry (10–12 Hz) (FAA), relative theta power (4–8 Hz), and relative low beta power (12–15 Hz) were used as the target EEG indices because these are known to get modulated according to an individual’s valence, relaxation level, and attention level, respectively [9,10,11]. The interquartile ranges (IQR) of these features were employed to measure their dynamic ranges [25]. 

For the Valence datasets, alpha powers (10–12 Hz) were first computed from each of the two channels (Fp1 and Fp2) using Matlab functions, periodogram and band power, provided by the signal processing toolbox of Matlab 2019a (The Mathworks, Inc., Natick, MA, USA). Then, the FAA was evaluated using the following equation:

(1) $$\mathrm{FAA}=ln\left(\frac{Alpha\;power\;at\;Fp1}{Alpha\;power\;at\;Fp2}\right).$$

For the relaxation and attention datasets, frontal theta (4–8 Hz) and low beta (12–15 Hz) powers were calculated, respectively, and then averaged across the two channels for all segments, including resting-state time periods. Relative frontal theta power (rFTP) and relative frontal low beta power (rFLBP) were evaluated by dividing the absolute theta and low beta powers by the total power (2–18 Hz). 

### 2.6. Extraction of Candidate EEG Predictors

For the artifact-free segments in the first RS-EEG dataset (resting-state 1 dataset), EEG band powers for seven frequency bands including delta (2–4 Hz), theta (4–8 Hz), alpha1 (8–9 Hz), alpha2 (10–12 Hz), alpha3 (8–12 Hz), low beta (12–15 Hz), and beta (15–18 Hz) were evaluated for each channel. We also computed the relative band powers by dividing the absolute band powers by the total power (2–18 Hz). The interhemispheric asymmetry of each band power was evaluated using *ln*{(band power at Fp1)/(band power at Fp2)}.

For each participant, the average and IQR of the above quantitative EEG (qEEG) parameters were evaluated over all the artifact-free segments in the RS-EEG dataset. Finally, 112 candidate EEG predictors were extracted from each participant’s RS-EEG dataset, which were combinations of eight frequency bands (seven bands and a full band), seven qEEG values (absolute and relative powers at Fp1 and Fp2, and their means; and the interhemispheric asymmetry of each band power), and two statistical indices (average and IQR).

### 2.7. Regression Models and Selection of Optimal EEG Predictors

In this study, various regression methods, such as multiple linear regression (MLR) [26], tree regression (TR) [27], ensemble bagged tree regression (ebTR) [28], support vector machine regression (SVMR) [29], kernel support vector machine regression (kSVMR) [30], and Gaussian regression process (GPR) [31] were used to construct a predictive model relating the RS-EEG predictors and IQRs of three EEG features. The performance of each regression model was evaluated using the LOOCV method in terms of the normalized root mean square error (*nRMSE*). The procedure to apply the LOOCV is as follows: (i) actual IQRs and RS-EEG predictors of all participants except a participant to be tested were used to construct a regression model; (ii) IQR of an EEG feature of the test participant was predicted with the participant’s RS-EEG predictors and the regression model constructed using other participants’ data; (iii) the procedures of (i) and (ii) were repeated until the IQRs of all participants were predicted; and (iv) nRMSE was computed using the actual and predicted IQRs of all participants as

(2) $$nRMSE=\;\frac{\sqrt{\frac{1}{N}\sum_{n=1}^{N}{\left(Actual\;IQR_{n}-Predicted\;IQR_{n}\right)}^{2}}}{Actual\;IQR_{max}-Actual\;IQR_{min}}$$

where *N, Actual IQR_max_*, and *Actual IQR_min_* represent the number of participants, the maximum value of actual IQRs, and the minimum value of actual IQRs, respectively.

While constructing a regression model predicting the IQR of a test participant’s specific EEG feature, the optimal combinations of candidate RS-EEG predictors were found using the forward sequential feature selection (FSFS) algorithm. The FSFS finds the best set of predictors by adding a new entry of predictors only when the nRMSE obtained by the training set reduces. Please note that the nRMSE here was not the nRMSE for the regression model evaluation but one that used as a criterion to select the optimal RS-EEG predictors.

## 3. Results

### 3.1. Interindividual Variability of EEG Features

**Figure 3:**
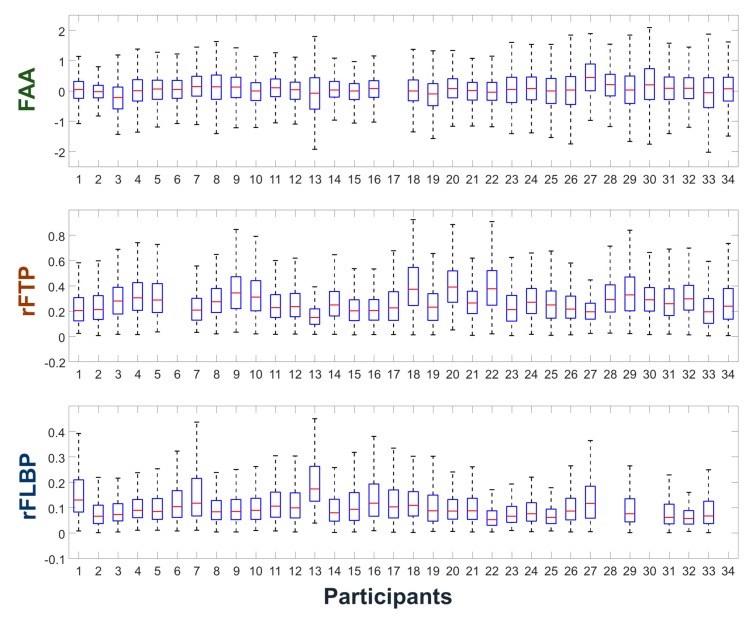
Boxplots showing the dynamic ranges of the individual participants’ frontal alpha asymmetry (FAA), relative frontal theta power (rFTP), and relative frontal low beta power (rFLBP) features. The vertical length of each box corresponds to the interquartile range (IQR) of each feature.

Figure 3 shows the dynamic ranges of individual participant’s FAA, rFTP, and rFLBP features having been widely employed to estimate user’s valence, relaxation level, and attention level in pBCI applications. Large interindividual variability of these features can be observed from the figure. For example, participant #2 shows much smaller dynamic ranges of FAA and of rFLBP than the average dynamic ranges. Participants #13 and #27 show small dynamic ranges of rFTP, which are approximately half of those of participants #18 and # 22. As seen from the boxplots, there are only a small portion of individuals who require additional calibration sessions for the individualization of EEG features. Please note that some participants’ datasets were missing for specific sessions (e.g., participant #17’s FAA dataset was missing) due to the technical problems during EEG recording.

### 3.2. Prediction of Dynamic Ranges of EEG Features

Table 1 shows the average nRMSE values for each EEG feature with respect to different regression models. For example, nRMSE of 0.1562 implies that the RMSE was approximately 15.62% of the actual IQR. The IQRs of FAA, rFTP, and rFLBP could be estimated with the lowest nRMSEs of 0.2323, 0.1820, and 0.1562, respectively, when SVMR, ebTR, and SVMR were employed, respectively. The linear regression model MLR showed the worst prediction performance among all the regression models. We also constructed a regression model with RS-EEG predictors that are the same as the three EEG features without using the FSFS algorithm. The results are presented in the second column of Table 1 (denoted by Baseline). To statically compare the nRMSEs between the baseline model without feature selection and the best regression models, one-tailed paired permutation tests were performed, with the total number of permutations being 10,000. Our results showed that *p*-values from the permutation tests in the prediction of IQRs of FAA, rFTP, and rFLBP were 0.3094, 0.3999, and 0.0313, respectively. Although statistical significance was reported only in the rFLBP feature, the machine-learning-based regression models with FSFS algorithm (SVMR in the estimation of FAA and rFLBP; and ebTR in the estimation of rFTP) showed better estimation performance than the baseline regression model without feature selection. The detailed information of the best regression models can be found in the Supplementary Information file attached to this manuscript.

**Figure 4:**
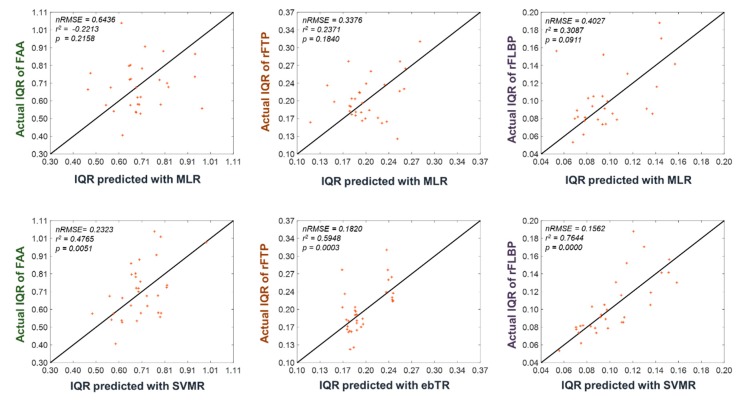
Scatter plots for the predicted and actual IQRs of three EEG features (FAA, rFTP, and rFLBP), obtained using multiple linear regression (MLR) (first row) and the best regression models (second row), respectively. Note that the IQR is the height of the box in the boxplots depicted in Figure 3. Each point (x, y) represents a participant’s predicted and actual IQRs after leave-one-participant-out cross- validation (LOOCV) application. Therefore, a point on the line y = x implies the perfect prediction of the dynamic range of the EEG feature. nRMSE, R-squared, and *p*-value values are provided at the top-left corner of each panel.

Figure 4 shows the scatter plots for the predicted and actual IQRs of the three EEG features, which demonstrates again the superiority of the machine-learning-based regression model (SVMR) in the prediction of the dynamic ranges of the EEG features. 

### 3.3. The Optimal Sets of RS-EEG Predictors

The optimal RS-EEG predictors that resulted in the best prediction accuracy were dependent on the training datasets and thus differed from one validation to another. The average numbers of the RS-EEG predictors selected for the prediction of the IQRs of FAA, rFTP, and rFLBP were 1.12 ± 0.33, 3.94. ± 1.00, and 3.18 ± 1.04, respectively. To identify the most frequently selected RS-EEG predictors, the top five frequently selected RS-EEG predictors are reported in Table 2. The names of the RS-EEG predictors take the form [Statistics (Mean or IQR)]-[Band Power Type (Relative or Absolute) or Asymmetry]-[Frequency Band]. For example, “IQR-Asym-Alpha-8-12” represents the IQR of the interhemispheric asymmetry of alpha (8–12 Hz) band powers. 

## 4. Discussion and Conclusions

In the implementation of practical pBCI applications, the small dynamic ranges of EEG features in some individuals have been an important issue and should be addressed. Introduction of calibration sessions for searching for individually customized EEG features has been suggested as a solution to tackle this problem; however, this process might not be necessary for most users of the pBCI applications, who show sufficiently large dynamic ranges of EEG features. In this study, we were able to predict each individual user’s dynamic ranges of the EEG features measuring valence, relaxation, and concentration levels (during the main task periods) from a short RS-EEG. The prediction results can be used to determine whether or not a specific user needs to undergo an additional calibration to find personalized EEG features. The procedure of predicting a user’s suitability for a specific pBCI application would be as follows: (i) collect 1-min resting EEG data from a user; (ii) extract RS-EEG predictors from the resting EEG data; (iii) predict the dynamic ranges of the EEG features using the machine learning model constructed previously (the parameters of the best regression models can be found in the Supplementary Information file attached to this manuscript); (iv) if the estimated dynamic range is less than a threshold (e.g., mean—standard deviation, where the mean and standard deviation are evaluated from the IQRs for all participants’ data), additional individualization process is conducted. 

We tested various machine learning-based regression models to predict the dynamic ranges of three widely used EEG features known to be associated with valence, relaxation, and concentration. Among the various regression models, SVMR with FSFS mostly showed the best performance in predicting the IQRs. The predicted IQRs of the EEG features were statistically significantly correlated with those predicted with SVMR. The excellent performance of SVMR might originate from its intrinsic nature, which is generally known to work more effectively in high dimensional spaces [32].

Although the selection of RS-EEG predictors was dependent on the training data used in each cross-validation, there were some RS-EEG predictors consistently selected in most LOOCV iterations. As shown in Table 2, firstly, the RS-EEG predictors that are similar to (or sometimes the same as) the three EEG features were most frequently selected to build the regression models. For example, (i) “IQR-Asym-Alpha-10-12” and “IQR-Asym-Alpha-10-12” were frequently selected for the prediction of the IQR of FAA; (ii) “IQR-Rel-Fp1-Theta-4-8” was frequently selected to predict the IQR of rFTP; and (iii) “IQR-Rel-Fp12-Beta-12-15”, “IQR-Abs-Fp1-Beta-12-15”, and “IQR-Rel-Fp2-Beta-12-15” were frequently selected for the prediction of IQR of FLBP. These findings seem to be natural considering that some previous studies reported that the amount of task-dependent modulation of specific EEG band power is tightly linked to the baseline EEG activity level recorded during resting state. For example, Blankertz et al. and Sannelli et al. [33,34] have shown in their motor imagery BCI studies that participants showing high activation of sensorimotor rhythm (SMR) in a relaxed recording session before the actual BCI session are likely to have stronger SMRs in the actual BCI session. In the meantime, some RS-EEG predictors whose frequency bands are quite different from those of the EEG features were also observed. These predictors include (i) IQR of delta power for the prediction of IQR of FAA; (ii) IQR of beta power for the prediction of IQR of FTP; and (iii) IQR of FRP for the prediction of rFLBP. Although a concrete justification is hard, tight cross-frequency couplings among alpha, delta, and beta frequency bands, such as delta–alpha cross frequency coupling [35,36] and theta–beta coupling [37], might be reflected here.

Although the present study demonstrated the possibility of predicting the dynamic ranges of EEG features for pBCI using short RS-EEG data, several issues still remain to be addressed in future studies. First, the selected RS-EEG predictors and the prediction performance can vary from one dataset to another because our machine learning-based techniques depend on the EEG data used for the training. Therefore, to generalize our results and further build a generic regression model to predict the dynamic ranges of EEG features, larger EEG datasets need to be employed. Second, validation of the regression model with totally new EEG datasets recorded by using different task paradigms would help to precisely evaluate the performance of our approach [38,39,40,41]. Third, the threshold value to determine whether a participant is appropriate for pBCI needs to be determined empirically considering the actual pBCI users’ satisfaction level. Fourth, our system predicted each individual’s “suitability” for pBCI but did not predict the individual‘s “performance” of the pBCI. It would be an interesting future topic to predict the performance of pBCI from resting-state EEG. Last, in this study, candidate EEG predictors were restricted only to the EEG band powers. If more EEG electrodes are available, EEG network measures can also be used to predict the dynamic ranges of EEG features [42,43], which we would like to pursue in our future study.

## Figures and Tables

**Table 1:** The *nRMSE* values for each EEG feature with or without the forward sequential feature selection (FSFS).

IQR of EEG Features	Baseline (No Feature Selection)	Machine Learning Algorithms with Feature Selection					
		MLR	TR	ebTR	SVMR	kSVMR	GPR
IQR of FAA	0.2761	0.6436	0.2504	0.3241	0.2323	0.5938	0.3143
IQR of rFTP	0.1879	0.3376	0.2147	0.1820	0.1867	0.2106	0.2290
IQR of rFLBP	0.1899	0.4027	0.2141	0.2118	0.1562	0.3077	0.1725

Note: MLR: multiple linear regression; TR: tree regression; ebTR: ensemble bagged tree regression; SVMR: support vector machine regression; kSVMR: kernel support vector machine regression; GPR: Gaussian regression process (GPR); IQR: Interquartile range. Baseline (no feature selection) represents a regression model constructed with the same EEG features used for the pBCI applications.

**Table 2:** Frequently selected RS-EEG predictors for each EEG feature.

IQR of EEG Features	Selected RS-EEG Predictors	Frequency of Selection
IQR of FAA	IQR-Asym-total	45.45
	IQR-Asym-Alpha-10-12	36.36
	IQR-Asym-Alpha-8-9	18.18
	IQR-Abs-Fp12-Delta-2-4	12.12
	n.r.	n.r.
IQR of rFTP	IQR-Rel-Fp1-Theta-4-8	93.94
	IQR-Abs-Fp12-Beta-15-18Hz	78.79
	IQR-Abs-Fp12-total	54.55
	IQR-Rel-Fp2-Beta-15-18	48.48
	IQR-Abs-Fp1-Beta-15-18	15.15
IQR of rFLBP	IQR-Rel-Fp12-Beta-12-15	84.37
	IQR-Abs-Fp12-Beta-15-18	62.50
	IQR-Abs-Fp1-Beta-12-15	43.75
	IQR-Rel-Fp2-Beta-12-15	28.13
	IQR-Rel-Fp12-Theta-4-8	12.50

Note: “n.r.” represents not reported.

## Supplementary Materials

The following are available online at https://www.mdpi.com/1424-8220/20/4/988/s1, Parameters of Regression Models.
