# Supplementary material for: Prediction of Individual User’s Dynamic Ranges of EEG Features from Resting-State EEG Data for Evaluating Their Suitability for Passive Brain–Computer Interface Applications
Source: Sensors (Basel). 2020 Feb 12;20(4):988. doi: 10.3390/s20040988 (PMC7071472; doi:10.3390/s20040988)
Supplement: Supplementary file 1 [file sensors-20-00988-s001.pdf]

Supplementary Information - Parameters of Regression Models

The parameters of regression models were uploaded at Figshare.com as MATLAB *.mat files.

Download the Regression Model MAT-Files (*.mat)

1. URL to download the model parameter files: https://figshare.com/s/a93b79d0b35e054810d8
2. Three regression model files (*.mat) are "mdl_FTP.mat (22.48 kB)", "mdl_FAA.mat(5.51 kB)", and "mdl_FLBP.mat (6.18 kB)". These files are freely downloadable.

Dependencies

1. Higher version of Maltab R2016b: https://mathworks.com/
2. Statistics and Machine Learning Toolbox: https://www.mathworks.com/products/statistics.html

Loading the Regression Models MAT-Files in Matlab

1. Make sure that the Regression Model Mat-Files are in the current path in Matlab
2. Load a Regression MAT-File by using the Matlab function, "load', as follows:

```
load('mdl_FAA.mat');
```

Predict the IQR of the EEG features

1. Recording resting EEG (RS-EEG) and prepare the following RS-EEG predictors:
   A. RS-EEG-Predictors for the prediction of the IQR of FAA
      - [IQR-Asym-Alpha-10-12, IQR-Asym-Alpha-8-9]

   B. RS-EEG-Predictors for the prediction of the IQR of FTP
      - [IQR-Rel-Fp12-Theta-4-8Hz]

   C. RS-EEG-Predictors for the prediction of the IQR of FLBP
      - [IQR-Abs-Fp12-Beta-15-18, IQR-Abs-Fp12-Beta-12-15, IQR-Rel-Fp2-Beta-12-15]
2. Predict the IQR of EEG features using the Matlab function, "predict", as follows:

```
Pred_IQR = predict(mdlSelectedTest,[0.428687343204822,0.442510355647657]);
```

Table. Hyperparameters that used to Train the Regression Models

IQR of EEG features	The regression models	Parameters
FAA	SVMR	*C* = 0.1594, ε = 0.0159, and δ = 1.0914
rFTP	ebTR	The total number of learning cycles = 22,<br>max number of splits = 1,<br>minimum number of leaf size = 4
rFLBP	SVMR	*C* = 0.0299, ε = 0.0029, and δ = 1.5813

C, ε, and δ represents penalty factor, insensitive loss coefficient, and width of the linear kernel function, respectively.
